# Distinguishing AKI from CKD: outcomes and characteristics of patients with abnormal serum creatinine and no known baseline

**DOI:** 10.1186/s12882-026-04758-8

**Published:** 2026-01-27

**Authors:** Esther Wong, Anna Casula, Rachael Hughes, Rosie Cornish, Kate Tilling, Nicholas M. Selby, James Medcalf

**Affiliations:** 1 UK Renal Registry, UK Kidney Association, Bristol, UK; 2 University of Bristol, Bristol, UK; 3 University Hospitals of Leicester NHS Trust, Leicester, UK; 4 University of Leicester, Leicester, UK; 5 University of Nottingham, Nottingham, UK; 6 University Hospitals of Derby and Burton NHS Foundation Trust, Derby, UK

**Keywords:** Acute kidney injury, Chronic kidney disease, Serum creatinine, Diagnosis, Clinical follow-up

## Abstract

**Background:**

Comparison of a patient’s abnormal serum creatinine result to an earlier value is fundamental to differentiating Acute Kidney Injury (AKI) from Chronic Kidney Disease (CKD), and is the first step in electronic AKI detection systems. For those patients in whom a baseline serum creatinine is unavailable, some systems generate a warning message to highlight the elevated serum creatinine but without distinguishing AKI from CKD (a “?AKI?CKD” warning). We aimed to determine demographic characteristics of this group, the proportion who had a first presentation of AKI, their clinical outcomes, and how these alert messages translate into subsequent biochemical testing and follow-up.

**Methods:**

We performed a retrospective cohort analysis of adult patients with serum creatinine testing at University Hospitals of Leicester during 2019. Using the NHS England AKI detection algorithm, we identified patients with AKI Warning Test Scores (WTS) and “?AKI?CKD” warnings. The “?AKI?CKD” cohort was classified as probable AKI, probable CKD, or no follow-up result, based on subsequent serum creatinine measurements. Survival (90-day and 1-year) was analysed with Kaplan–Meier methods.

**Results:**

Among 3,464 patients with “?AKI?CKD” warnings, 8.5% were probable AKI, 59.4% probable CKD, and 32.0% had no follow-up test. Probable AKI patients were younger (median age 71 versus 76 years) and more often hospitalised at warning time (56% versus 15%). One-year survival was lower in probable AKI (72%) compared to probable CKD (88%) or no follow-up (89%). Probable AKI survival was similar to AKI WTS stage 1 but better than stages 2–3. Extending baseline serum creatinine look-back to 426 days changed categorisation minimally (≤ 2%).

**Conclusions:**

These findings highlight that the major feature of the “?AKI?CKD” classification is not simply misclassification between AKI and CKD, but the variability of clinical response, with one-third of patients receiving no subsequent serum creatinine test. Most patients flagged as “?AKI?CKD” likely have CKD rather than AKI, and this, coupled with comparable outcomes of the probable AKI group to early-stage AKI, suggests minimal missed population-level AKI detection. However, one-third lacked follow-up testing, highlighting missed opportunities to identify CKD.

**Clinical trial number:**

Not applicable.

**Supplementary Information:**

The online version contains supplementary material available at 10.1186/s12882-026-04758-8.

## Key learning points

• One-third of flagged patients have no repeat serum creatinine test, which is a potential missed opportunity for early diagnosis.

• Patients with an abnormal serum creatinine result but no baseline most often have undiagnosed or existing chronic kidney disease (CKD), rather than acute kidney injury (AKI).

• Patients with probable AKI in this group have higher short-term mortality and are more often hospitalised than those with probable CKD.

**Supplementary Information:**

The online version contains supplementary material available at 10.1186/s12882-026-04758-8.

## Abstract (significance)

What was known

This study adds

• Missing baseline serum creatinine slows down automated AKI detection and differentiation from CKD.

• AKI detection algorithms commonly generate a “?AKI?CKD” flag with elevated serum creatinine to avoid misclassification when no prior serum creatinine exists.

• The proportion of AKI versus CKD in this flagged group, and their clinical outcomes, were not previously well established.

• Only about 8.5% of “?AKI?CKD” patients meet criteria for probable AKI; the majority are probable CKD.

• Probable AKI patients have higher early mortality and hospitalisation rates than probable CKD.

• One-third received no follow-up serum creatinine test, identifying gaps in recommended care pathways.

**Supplementary Information:**

The online version contains supplementary material available at 10.1186/s12882-026-04758-8.

## Potential impact

Supports maintaining “?AKI?CKD” as a separate alert category to limit AKI overdiagnosis.

Stresses the importance of prompt repeat serum creatinine testing in uncertain kidney function cases.

Identifies the need for targeted clinical follow-up systems to address missed testing opportunities that will increase CKD detection, which is particularly important with the expanding therapeutic landscape for these patients.

**Supplementary Information:**

The online version contains supplementary material available at 10.1186/s12882-026-04758-8.

## Background

Measurement of serum creatinine is a very common test in routine clinical care, and the interpretation of an abnormal result is significantly enhanced if there is an earlier result in the same patient to compare it to (ideally within the previous 12months). In the absence of a contemporary result for comparison, differentiating acute kidney injury (AKI) from Chronic Kidney Disease (CKD) becomes much harder. Often, a rapid repeat measurement is required, and even then, there could be concerns that a significant AKI was undetected or diagnosis was delayed, highlighting a critical operational challenge impacting patient safety.

AKI is a rapid decline in kidney function over a period of hours or days and is associated with poor health outcomes, including increased mortality [1–3]. AKI can be detected by an increase in serum creatinine with respect to an individual’s baseline value, a decrease in urine volume, or both [4]. Early detection and intervention may lead to better outcomes [5–7]. In 2014, the National Health Service (NHS) in England mandated the national implementation of a standardised AKI detection system [8]. Full details are provided in Supplementary Information (S1), with AKI Warning Test Scores (AKI WTS) generated for elevated serum creatinine values that correspond to AKI stages 1, 2, or 3 [4]. Very similar AKI detection algorithms exist in other clinical systems, and like the NHS algorithm, are generally based on KDIGO guidance [4].

In cases in which there are no previous values to use as a baseline, the NHS algorithm generates a text message to highlight the abnormal result and indicate that this may indicate AKI or CKD (termed “?AKI?CKD warning“). We aimed to determine demographic characteristics of this group, the proportion who had a first presentation of AKI, their clinical outcomes, and how these alert messages were associated with subsequent biochemical testing and follow-up. We also examined whether patients lacking a baseline serum creatinine and generating ?AKI?CKD warnings represented missed opportunities for AKI care.

Previous authors have highlighted that there is no standard approach to estimating baseline serum creatinine when no preadmission value is available [9], with surrogate baseline choices markedly affecting AKI incidence and prognosis estimates. For example, Cooper et al. [10] demonstrated that back-calculating baseline serum creatinine using an assumed eGFR of 75 ml/min/1.73 m² underestimated AKI incidence by over 50% in a young Southeast Asian infection cohort, whereas in other scenarios this approach can misidentify CKD as AKI. Unlike these studies, which attempt to impute missing baselines, the NHS England algorithm [11] deliberately avoids estimating a baseline, instead issuing a separate ?AKI?CKD warning when no prior creatinine is available.

The primary objective of this study was to describe the characteristics and outcomes of patients generating ?AKI?CKD warning test scores within the NHS AKI detection system, to examine their subsequent biochemical testing and follow-up, and to explore whether this group may represent missed opportunities for timely AKI recognition and management.

## Methods

This retrospective cohort study analysed data from adults with serum creatinine measurements performed by the University Hospitals of Leicester NHS Trust (UHL) during 2019. The year 2019 was specifically chosen to avoid potential effects introduced by the COVID-19 pandemic, thus providing a cleaner baseline period for analysis. This approach ensures that observed patterns and outcomes were not influenced by the major healthcare disruptions and changes in testing and admission practices during the pandemic era. UHL is a tertiary acute care provider to a diverse population of 1 million people. The study included all patients with AKI WTS (stages 1, 2, and 3) and those with ?AKI?CKD warnings. Patients on dialysis and episodes related to childbirth were excluded. The flow of patients through the study, including application of inclusion and exclusion criteria and classification into AKI Warning Test Score and ?AKI?CKD alert groups, is shown in Supplementary Figure S1b.

Demographic information, subsequent serum creatinine measurements, and patient outcomes were analysed over three time periods: 0–14 days, 14–90 days, and 91–365 days post-warning. AKI WTS was triggered according to the NHS England standardised algorithm, which defines AKI as an increase in serum creatinine of ≥ 26 µmol/L within 48 h, or an increase to ≥ 1.5 times baseline serum creatinine. The baseline creatinine is defined as the lowest value in the last 7 days, or as the median of values from the preceding days 8-365 depending on the availability of prior results. If no baseline exists within 365 days, the algorithm cannot classify AKI, and an ?AKI?CKD warning is issued when serum creatinine exceeds the upper reference limit.

**Fig. 1:**
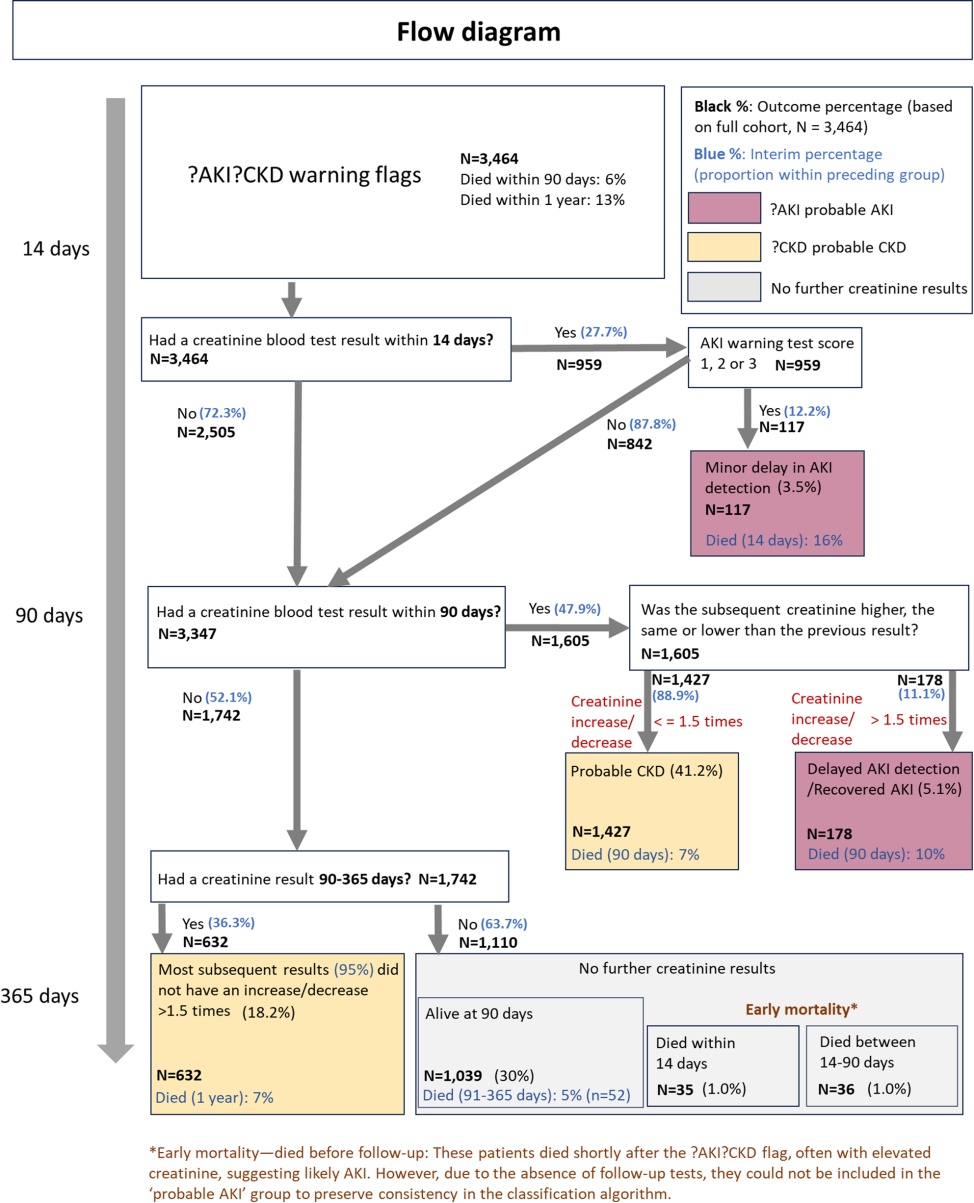

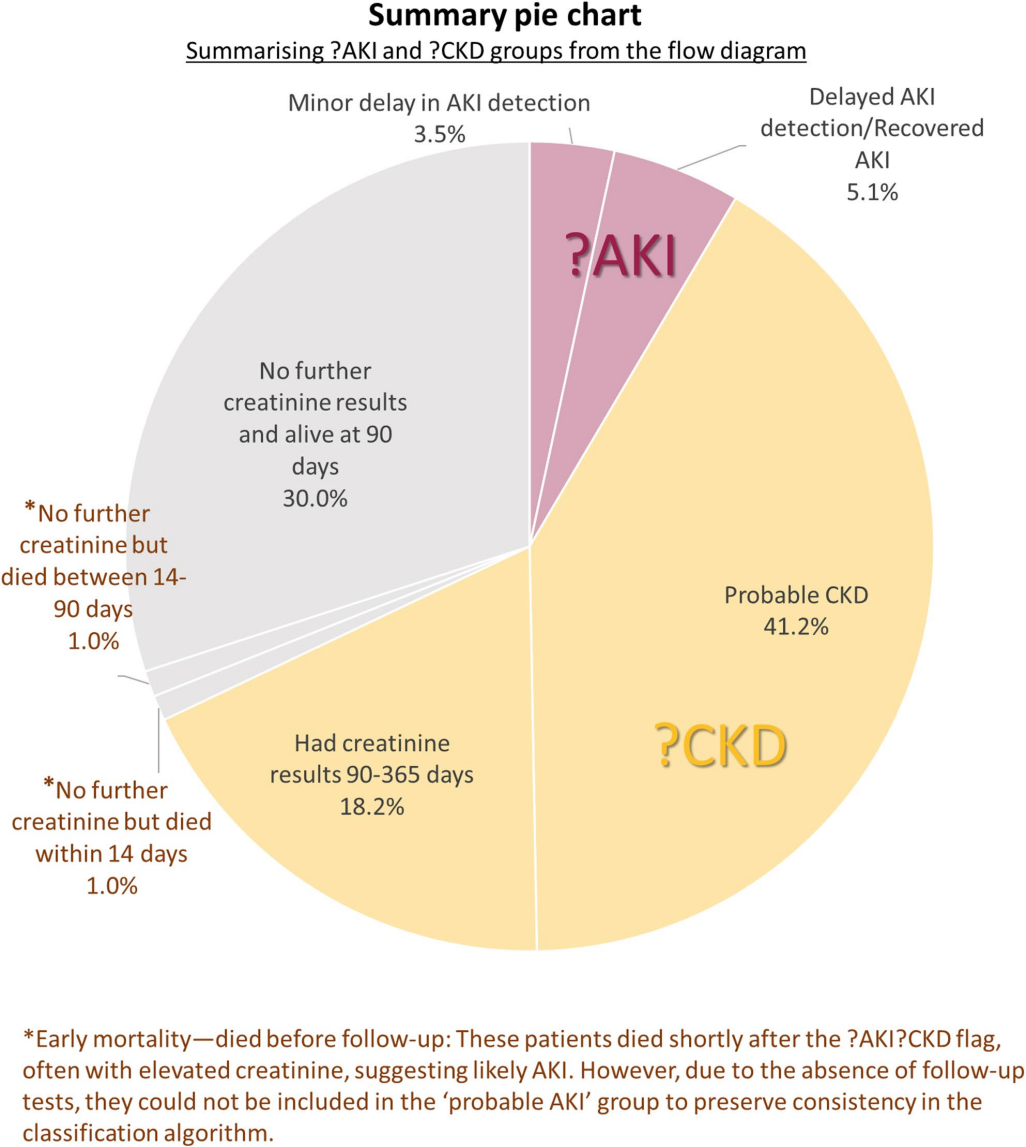
**a** Flow diagram for classification of patients with concurrent ?AKI?CKD warning Flags using sequential serum creatinine monitoring. The three resulting groups (?AKI, ?CKD, and no further serum creatinine result) are colour‑coded, and the same colours are used in **b** to indicate the corresponding group proportions. In this and subsequent figures and tables, “creatinine” refers to serum creatinine. **b** Summary pie chart showing the proportions of patients in each classification group (?AKI, ?CKD, no further serum creatinine result) using the same colour scheme as in **a**

We used subsequent serum creatinine results to classify those with ?AKI?CKD warnings into three groups: probable AKI, probable CKD and no further serum creatinine result (Fig. 1 and more details in S2). Probable AKI was defined as a subsequent serum creatinine value within 14 days that generated an AKI WTS, or a subsequent serum creatinine value within 90 days that either increased or decreased by ≥ 1.5 times the initial value. Probable CKD was defined as subsequent results within 90 or 365 days that were stable (i.e. changed < 1.5 times the initial value).

Dialysis patients were excluded by linkage with the UK Renal Registry (UKRR), where all individuals receiving chronic kidney replacement therapy are routinely identified on the national database. Patient records with serum creatinine values outside the laboratory analysable range, or defined as extreme results (e.g. less than 20 µmol/L or greater than 1500 µmol/L), were excluded from all analyses to minimise misclassification and analytical error.

Kaplan-Meier was used to evaluate 90-day and one-year survival. Differences in survival curves were assessed using the log-rank test. Statistical analyses were performed using SAS software, version 9.4.

Postcodes collected by UHL were mapped to Index of Multiple Deprivation (IMD) scores for England, and these scores were assigned to deprivation quintiles using national thresholds for comparative analyses.

## Results

During 2019 there were 9,805 patients with AKI WTS and 3,464 patients with ?AKI?CKD warnings. Figure 1a includes a pie chart 1b to show the distribution of final outcomes for patients with ?AKI?CKD warnings. The largest proportion of patients (59.4%) had probable CKD (41.2% with repeat serum creatinine within 90 days and 18.2% between 91 and 365 days). Only 8.5% were categorised as probable AKI (3.4% with AKI WTS within 14 days, and 5.1% with an increase or decrease in serum creatinine within 90 days). A substantial proportion of patients (32.0%) had no further serum creatinine results.

Demographics are summarised in Table 1, more details are provided in Supplementary Table S3. In those with AKI?CKD warnings, there were more males in the probable CKD group as compared to the probable AKI (77% versus 66%). Patients with probable CKD had a higher median age (76 years, IQR: 64–84) than those with probable AKI (71 years, IQR: 59–84). For those in the AKI Warning Test Score (WTS) group, the median age was 73 years (IQR: 59–83). In both ?AKI?CKD warning and AKI WTS groups, almost half came from the fourth and fifth least socially deprived quintiles.

**Table 1:** Demographic characteristics of patients with ?AKI?CKD and AKI warning test scores (AKI WTS)

	?AKI?CKD				AKI peak Warning Test Score			
	Total	?AKI	?CKD	No further creatinine	Total	Stage 1	Stage 2	Stage 3
	*N*	Col %	Col %	Col %	*N*	Col %	Col %	Col %
**Sex**								
F	847	31	26	21	5017	54	50	39
M	2615	69	74	79	4788	46	50	61
Missing	2	0	< 1%	< 1%				
**Age**								
18 to 39	203	10	4	8	899	11	6	5
40 to 64	708	30	19	20	2244	23	22	25
65 to 74	749	20	23	20	1970	20	21	22
75 to 84	959	21	28	28	2589	26	29	27
85 or over	845	19	25	24	2103	21	23	21
**Season**								
1. Spring	746	24	23	19	2432	25	26	24
2. Summer	982	27	28	30	2532	27	23	25
3. Autumn	910	27	24	31	2230	22	24	22
4. Winter	826	21	26	21	2611	26	26	29
**Deprivation quintile**								
1 - Most deprived	448	14	13	13	1635	16	17	18
2	678	21	20	18	1953	20	20	21
3	610	21	18	16	1814	18	19	20
4	871	22	25	27	2310	24	23	22
5 - Least deprived	842	20	24	25	2093	22	22	20
Missing	15	1	< 1%	1				
**Total**	3464	-	-	-	9805	-	-	-

*The peak AKI WTS was the highest warning score recorded in 2019

**Fig. 2:**
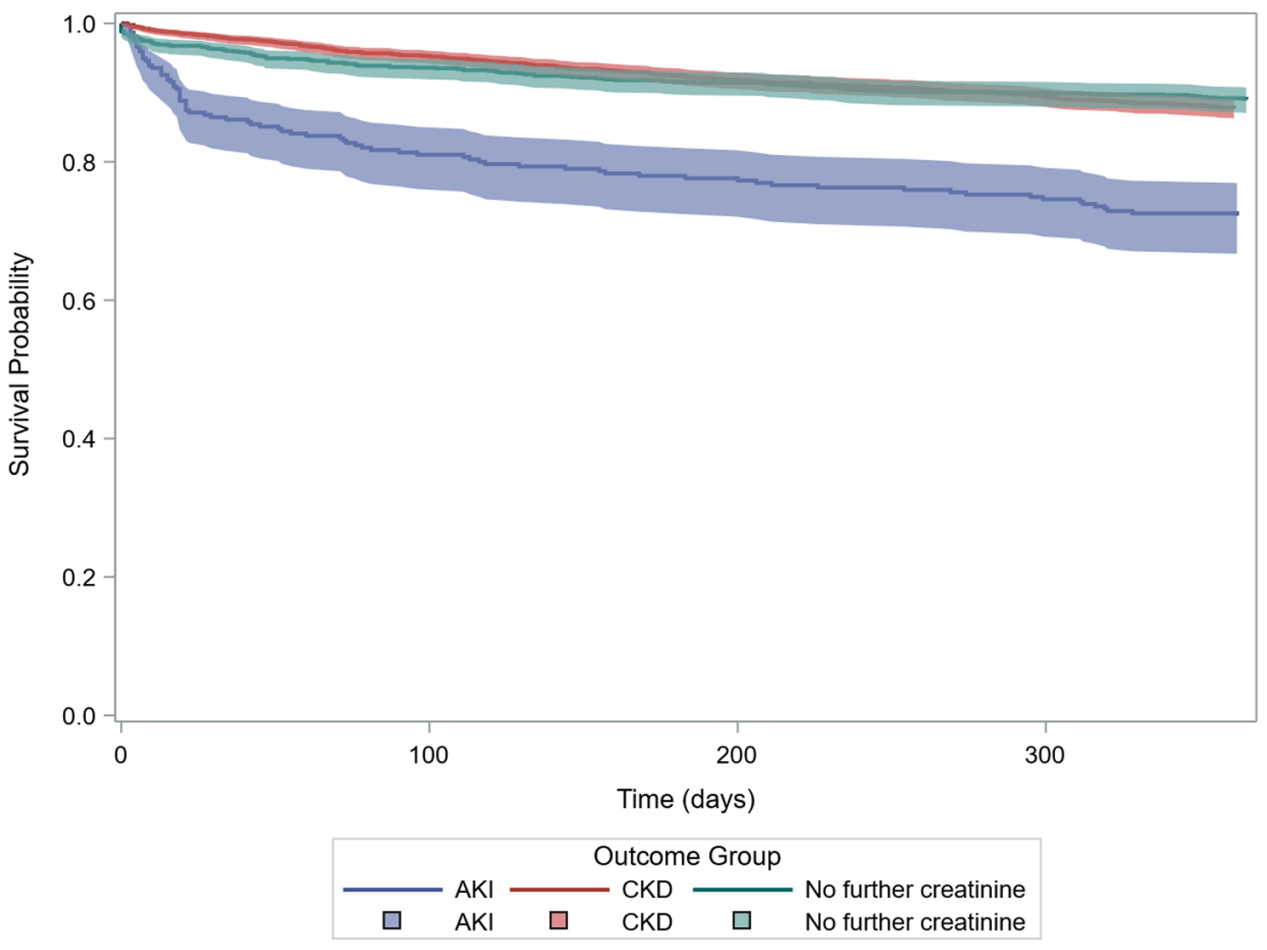
One-year survival of patients (*N* = 3464) who had ?AKI?CKD warnings by probable AKI, probable CKD and no further serum creatinine results groups. Survival differences were statistically significant (log-rank test: χ² = 71.4, df = 2, *p* < 0.0001)

Survival differed markedly between groups (Fig. 2). One-year survival was approximately 90% for both probable CKD and those without further serum creatinine results, but was lower in probable AKI (72%), with excess mortality in the first 90 days. Differences between groups at one year were statistically significant (Log-Rank *p* < 0.0001). Among patients stratified by AKI WTS (Fig. 2, Table S4), survival declined progressively with increasing stage severity, with one-year survival dropping from 72% in stage 1 to 56% in stage 2 and 54% in stage 3; survival of the probable AKI group was comparable to that of AKI WTS stage 1.

Hospitalisation patterns (Table S5a, S5b) also varied. Probable AKI patients were hospitalised at the time of the ?AKI?CKD warning more often than probable CKD (56% versus 15%), and 82% of probable AKI hospitalised within 90 days versus 36% for probable CKD. Corresponding figures for those with no further serum creatinine results were 9% and 15% respectively. The 14-day and 90-day figures represent any hospitalisation occurring in those timeframes, including continuation of the index admission, a readmission, or a first-time admission post-flag; they are not strictly cumulative from the flag-time hospitalisation rate, but reflect the broader hospital burden over time. Among all patients who were in hospital at the time of the ?AKI?CKD warning, 29% were classified as probable AKI (165/573), 54% as probable CKD (312/573), and 17% had no further serum creatinine result (96/573) (Table S5c). In contrast, the majority (60% and 38%, respectively) of those who remained out of hospital for at least 14 days after the alert were probable CKD or had no further serum creatinine, while only 2% were probable AKI (59/2,558).

We assessed the impact of extending the time-period to select baseline serum creatinine values beyond the NHS England algorithm to include 365 to 426 days (12 months + 2 months allowance for delayed annual reviews; see Supplementary 1). This had minimal impact as 98% of cases maintained their original categorisation.

To assess whether unconnected laboratory systems led to missing baseline serum creatinine, we linked ?AKI?CKD cases to the AKI-MPI dataset, and found just 5% had AKI alerts recorded by other laboratories in 2019.

## Discussions and conclusions

We investigated the characteristics and outcomes of patients with elevated serum creatinine measurements who lacked prior baseline measurements, in whom it is difficult to differentiate AKI from CKD based on serum creatinine values alone. Various approaches to this situation have been proposed, including applying AKI criteria using an estimated baseline serum creatinine that is reverse calculated from an eGFR of 75 ml/min/1.73m^2^ [12], but this risks misclassifying patients with CKD as having AKI. The NHS England algorithm avoids this by only flagging the abnormal serum creatinine without attempting to classify whether the result is consistent with AKI or CKD. Our findings support this approach as less than 10% of those with ?AKI?CKD were categorised as having probable AKI, and 40% of these were identified by the algorithm as AKI within the following days. Therefore, the potential impact of missed AKI due to absent baseline serum creatinine is likely minimal at the population level, although this may vary in other healthcare systems in which biochemistry testing is more fragmented.

Males predominated in ?AKI?CKD cases (2,615/3,464, 75% vs. 4,788/9,805, 49% in AKI WTS), and this excess was evident across ?AKI, ?CKD and no‑follow‑up subgroups. This misclassification reflects males’ higher baseline creatinine, potentially causing single measurements to more frequently exceed age-sex-specific laboratory reference ranges [1]. The WTS’s relative-change approach showed balanced sex distribution. The greater representation of less deprived individuals within the ?AKI?CKD group may reflect local population characteristics and differential patterns of healthcare use, such as a higher frequency of testing in less deprived areas. This observation may also relate to catchment demographics rather than a true disparity in disease incidence, and further evaluation in broader, multi‑centre cohorts would be valuable.

It is important to highlight that our work tests a complementary hypothesis focused on the clinical and operational consequences of missing baseline serum creatinine values rather than solely on algorithmic imputation. While prior studies have concentrated on methods to estimate baseline serum creatinine to enhance AKI detection, our study evaluates whether flagging patients with no baseline serum creatinine as ?AKI?CKD leads to missed AKI care. Our findings demonstrate that only a minority of these patients are subsequently classified as probable AKI, many of whom were promptly identified with repeat serum creatinine testing. This adds conceptual depth by linking biochemical algorithm outputs to real-world care pathways and outcomes, an area less explored in previous research.

Most patients with an ?AKI?CKD warning likely had CKD, with the lack of previous serum creatinine and eGFR testing suggesting that at least some of these cases were previously undiagnosed. This group, and those without further serum creatinine tests, had substantially lower mortality rates compared to those with probable AKI. Further, a much higher proportion of those with probable AKI were hospitalised at the time of the ?AKI?CKD result, supporting our categorisation. The results also demonstrate that ?AKI?CKD warnings are disproportionately represented among patients who are in the hospital at the time of ?AKI?CKD warning or require admission soon after, highlighting that this subgroup captures individuals with higher acute morbidity and concurrent illness. This supports the view that inpatient triggers are more closely linked to acute illness episodes, while outpatient alerts often indicate progressive CKD or stable chronic disease entering surveillance, thus confirming anticipated clinical phenotypes within the AKI warning algorithm. Our results indicate that only a small proportion of ?AKI?CKD warnings represented possible missed AKI, and these cases had limited clinical impact. The findings therefore do not suggest a need for intervention or system change to address missed AKI cases in this setting, given the minimal clinical impact observed.

However, 32% of patients with ?AKI?CKD warnings had no follow-up creatinine testing, suggesting that some alerts were not acted upon. This represents a broader care gap in CKD recognition and monitoring, beyond the AKI algorithm’s scope. This care gap is more related to CKD detection and monitoring, representing a potential target for future primary care quality improvement efforts beyond the AKI algorithm itself. Patterns of hospitalisation and survival in these patients were very similar to those with probable CKD, and we can infer that only a few represented unrecognised AKI cases. Nevertheless, repeat testing remains essential to distinguish between AKI and CKD, and earlier testing may have enabled timelier AKI diagnosis in the small group with probable AKI. Importantly, this represents a missed opportunity for detecting previously undiagnosed CKD; CKD detection is becoming increasingly important with the expanding repertoire of effective therapies to slow its progression and reduce cardiovascular risk. The lower mortality in this subgroup suggests many may not have required urgent evaluation, although this cannot be confirmed from our data. Possible reasons for no follow-up were likely due to clinical judgement that further testing was unnecessary, patient non-attendance, or missed diagnostic opportunities. These mechanisms cannot be distinguished in this study and would need to be explored in future research or audit before considering any specific service‑level intervention.

Thus, while prompt repeat testing is essential for distinguishing AKI from CKD and preventing short-term harm, the longer-term opportunity lies in detecting undiagnosed CKD, which has become increasingly important with the expanding availability of effective therapies.

Higher mortality and hospitalisation among probable AKI reinforce the clinical importance of distinguishing between acute and chronic kidney dysfunction. Probable AKI had similar 90-day and 1-year survival rates to those with definite AKI stage 1 (based on AKI WTS), while survival was worse with definite, severe AKI (AKI WTS Stage 2/3). We speculate patients with an ?AKI?CKD warning and probable AKI may have experienced milder AKI or, due to the imperfect categorisation of probable AKI, some could have had underlying CKD.

Missing baseline serum creatinine values are a recognised limitation in AKI detection. Possible causes include fragmented data if patients have blood tests analysed in different laboratories with separate laboratory IT systems, due to patients travelling to receive healthcare or living near the borders of catchment areas. However, only 5% of patients were tested outside the Leicester laboratory, indicating this was not a major issue; for most, the flagged result was likely their first serum creatinine test.

Extending the lookback window to 426 days identified < 2% additional AKI cases, supporting the NHS England algorithm’s current 365-day timeframe and its effectiveness in capturing most clinically significant cases given the annual primary care monitoring.

Surrogate baseline creatinine methods risk misclassification bias by under- or overestimating AKI incidence [13], potentially missing diagnoses or over-diagnosing CKD with associated costs. The NHS England algorithm mitigates this by flagging patients without measured baselines separately, avoiding CKD misclassification as AKI; our data (3,464 missing baselines) suggest minimal net impact on AKI identification. While prior studies report up to 50% missing rates necessitating surrogates [14, 15] our results likely reflect strong local laboratory integration, noting that national generalisability may vary due to variable data availability.

This study has limitations. First, it was conducted within a single regional health system with integrated laboratory data, which may limit wider generalisability. Second, classification of probable AKI and CKD was based on biochemistry and timing rather than full clinical review, introducing possible misclassification. Third, data on comorbidities, treatments, and care processes were not available.

In summary, most patients identified by the NHS England AKI algorithm as having a high serum creatinine with no previous values probably do not have AKI. This supports the current practice of identifying this group separately to AKI Warning Stage Results, but we have identified potential gaps in care with a substantial proportion not having repeat serum creatinine checks as recommended in AKI and CKD guidelines. This is an area that could be targetted within primary care CKD initiatives that aim to improve CKD detection and increase prescription of guideline-recommended therapies.

## Supplementary Information

Below is the link to the electronic supplementary material.

Supplementary Material 1

## Data Availability

The data underlying this study were obtained from the UK Renal Registry (UKRR), linked with data from the University Hospitals of Leicester NHS Trust and Hospital Episode Statistics (HES). Access to these data is restricted. Researchers interested in UKRR data should apply through the UK Renal Registry’s data application process. Further details about the application process and access conditions can be found at [[https://www.ukkidney.org/audit-research/how-access-data/ukrr-data] (https://www.ukkidney.org/audit-research/how-access-data/ukrr-data)]. Researchers interested in accessing these data should submit a formal application, which will be reviewed following the UK Renal Registry’s governance and ethical approval policies. Access to the University Hospitals of Leicester NHS Trust data requires a separate application to their trust. Access to HES data is subject to approval by NHS England and a data sharing agreement between NHS England and the applicant’s institution. The authors do not have permission to share the linked dataset directly.

## Declarations

### AI use

Artificial intelligence tools (Perplexity AI, Grammarly) were used solely to assist with grammar, language, and stylistic editing during manuscript preparation. No generative AI tools were used for data analysis, interpretation, or substantive content generation. All authors reviewed and approved the final text, and accept responsibility for its content.
