# Supplementary material for: Distinguishing AKI from CKD: outcomes and characteristics of patients with abnormal serum creatinine and no known baseline
Source: BMC Nephrol. 2026 Jan 27;27:126. doi: 10.1186/s12882-026-04758-8 (PMC12918265; doi:10.1186/s12882-026-04758-8)
Supplement: Supplementary file 1 — Supplementary Material 1 [file 12882_2026_4758_MOESM1_ESM.docx]

# Supplementary 1 Detailed methodology

## UK Renal Registry (UKRR)

The UKRR is a charity hosted within the UK Kidney Association (UKKA) with permission to process patient identifiable information of people with kidney disease in the UK. In 2015, the National Health Service (NHS) in England mandated a standardised AKI detection and warning system based on all routine measurements of serum creatinine (NHS England 2014). As well as reporting a warning of possible AKI to clinicians on the basis of a change in serum creatinine, the results of those warnings are also required to be sent for national comparison to the UK Renal Registry (UKRR). University Hospital of Leicester NHS Trust(UHL) (along with almost all other laboratories in England treating patients in the NHS) routinely sends the UK Renal Registry (UKRR) identifiable patient-level information monthly. This data includes all the AKI Warning Test Scores (WTS, stages 1, 2 and 3) generated in the UHL Laboratory Information Management System (LIMS).

## NHS England AKI Algorithm

The standardised algorithm for generating AKI WTS (Figure S1)(NHS England 2014) requires a previous serum creatinine concentration measurement – referred to as a baseline - to detect acute changes in kidney function. This algorithm compares the current serum creatinine level to the most recent baseline within the past 365 days. This baseline is interpreted differently depending on whether it is from the previous 0-7 or 8-365 days, reflecting short-term vs longer-term changes. If the laboratory has no baseline serum creatinine measurement within the last 365 days then no comparison can be made, the algorithm cannot determine whether the current result represents a significant change, therefore, no AKI WTS can be generated. In that circumstance, and if the current serum creatinine measurement is above the upper limit of the reference interval, then a warning is issued along with the serum creatinine result highlighting that either AKI or chronic kidney disease (CKD) is possible, and a repeat measurement should be considered. This warning is labelled as ?AKI?CKD.

###
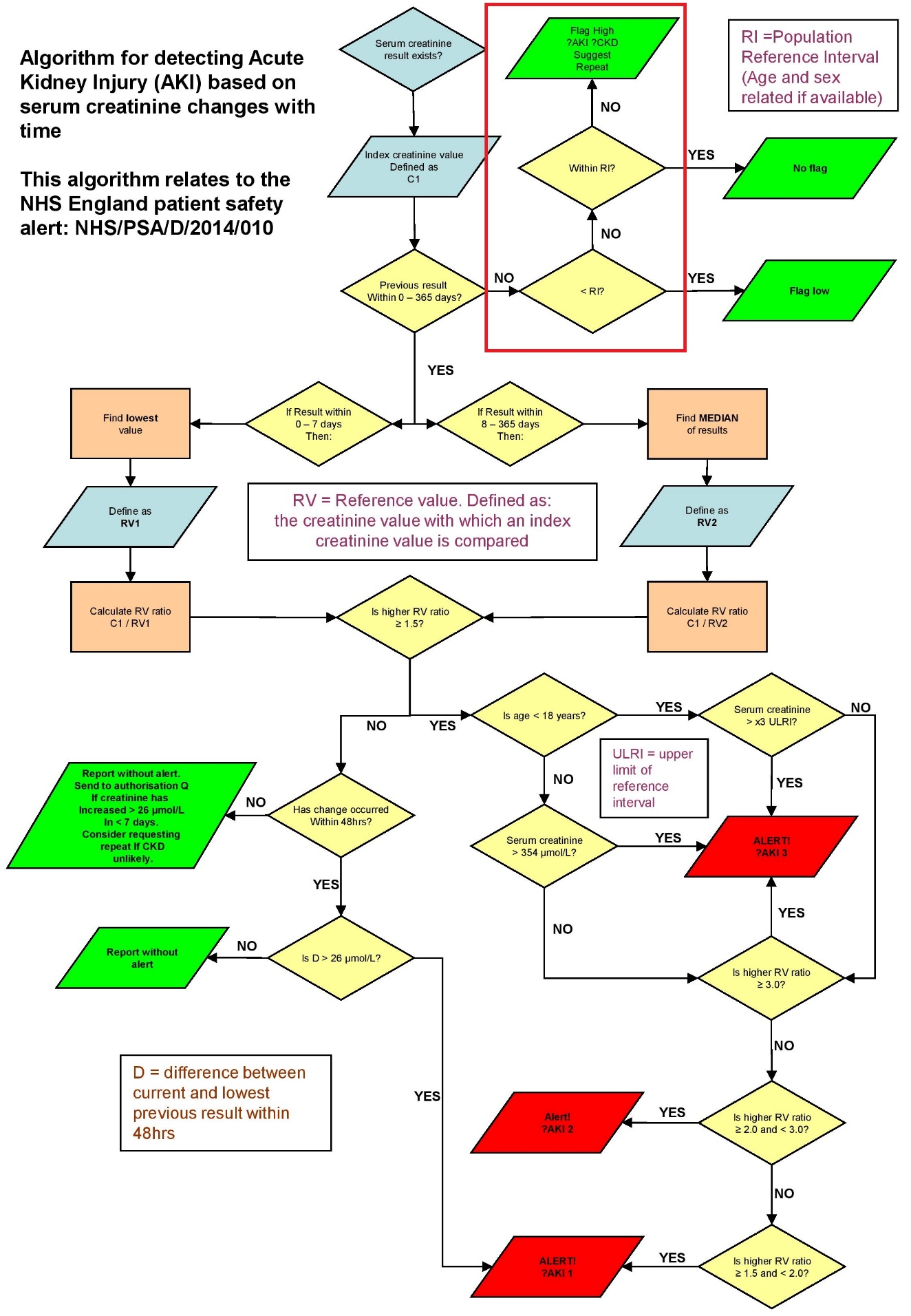
Figure S1a. NHS England AKI detection algorithm, reproduced under the Open Government Licence v3.0. Source: NHS England 2014b. The red square denotes patients with ?AKI?CKD warnings.

The number of people in the UK who have a creatinine test result but no baseline measurement within the past 365 days is not currently known. There are several possible reasons why a patient might lack a baseline measurement: for example, a healthy individual may never have needed a creatinine test before; someone with known CKD might not have had one in over a year; or a previous test may have been performed by a different provider using a separate results system, particularly in urban areas with multiple healthcare trusts.

It is also unclear what typically follows an elevated serum creatinine result — for example, how many patients receive a follow-up test within a few days, how many cases represent the first presentation of AKI, and how many reflect stable CKD.

## Data Extraction

To investigate patients with a serum creatinine result above the upper limit of the reference interval but no baseline creatinine measurement within the preceding 365 days, University Hospitals Leicester (UHL) provided the UK Renal Registry (UKRR) with additional warning flags labelled as "?AKI?CKD." These were added to the standard dataset of AKI Warning Test Scores (WTS), which are generated nationally using the NHS AKI algorithm. All patients in England who triggered either an AKI WTS or a ?AKI?CKD warning during 2019 were included in the combined dataset for analysis.

For each warning result, UHL supplied basic demographic information including patient age, sex, and postcode. Postcode was used to derive the Index of Multiple Deprivation (IMD). No other clinical information was available for the full cohort.

In addition to collecting AKI WTS into AKI Master Patient Index (AKI-MPI), the UKRR also maintains national records of all patients receiving chronic kidney replacement therapy (KRT). UKRR routinely links these data to Hospital Episode Statistics (HES), which provides information on all NHS hospital admissions. Mortality data were sourced from HES-ONS mortality data (NHS Digital 2023), which combines HES data with death registrations from Office for National Statistics (ONS), enabling analysis of hospitalisation, receipt of acute or chronic KRT, and mortality outcomes.

## Analytical methods

Subsequent re-checking of a patient serum creatinine measurement, comparison between measurements, and patient outcomes were considered at three time periods after the ?AKI?CKD warning– 0-14 days, 14-90 days and 91-365 days. This was to fit with standard recommendations to recheck an abnormal result within 14 days if AKI is a possibility, within 90 days to fit with a standard timeframe for reporting mortality, and within one year for the standard recommendations for rechecking creatinine with stable CKD.

Comparison was made regarding the proportion of people present in the UHL cohort who had had a serum creatinine check in any other England laboratory system to investigate the impact of any cross-boundary patient or blood test flow. Comparison was made using all patients in England reported to the UKRR with a WTS during 2019 to determine how many of those patients had a WTS from another England laboratory during the same year.

Finally, for all patients without a baseline creatinine measurement in the preceding 365 days the presence of an earlier possible baseline between 12 months and 14 months was also considered. The extension to 426 days (12 months + 2 months) was applied to account for patients who might not attend follow‑up blood testing exactly at one year; a two‑month allowance was considered a reasonable timeframe for delayed annual reviews. To evaluate the potential impact of extending the baseline window, we re-applied the AKI algorithm to all creatinine results from March to December 2019, using both the standard 365-day and an extended 426-day baseline window. We then compared the resulting peak AKI stage classifications to assess whether broader historical data could reduce the number of ?AKI?CKD flags and influence AKI detection.

## Exclusion criteria

**Patients on dialysis**

The cohort was linked to the UKRR database of all patients receiving chronic KRT to allow exclusion of any results from patients receiving chronic KRT

**Paediatric patients**

Patients under 18 years were excluded.

**Invalid creatinine results**

- Missing collection dates were excluded
- More than 10 days between the processing date and the collection date were excluded
- Extreme creatinine values were excluded
- Missing creatinine values were excluded
- Creatinine processing dates before the date of birth of the patient were excluded

**Hospital childbirths**

Physiological changes during pregnancy results in most pregnant women having a decrease in serum creatinine. The rapid physiological changes following delivery of the baby and common rise in serum creatinine not due to AKI means that patients with serum creatinine measurements taken during a childbirth admission were excluded.

## CONSORT

**Figure S1b** delineates the patient selection process from the Leicester laboratory creatinine database in 2019. From an initial dataset of 311,056 creatinine results transferred to the UKRR server, the AKI algorithm (Figure S1a) was applied using creatinine results from 2018 as a baseline to generate AKI WTS and identify potential ?AKI?CKD warnings. After applying exclusion criteria, including removal of dialysis unit results, paediatric cases, and incomplete data, the final cohort were categorised into two distinct groups: 3,464 patients with ?AKI?CKD warnings and 9,805 patients with AKI WTS across stages 1, 2, and 3 through the NHS England AKI algorithm.

### Figure S1b: CONSORT diagram of patient selection and classification for AKI Warning Test Scores and ?AKI?CKD warnings/flags from Leicester laboratory serum creatinine data. In this and subsequent figures and tables, “creatinine” refers to serum creatinine.


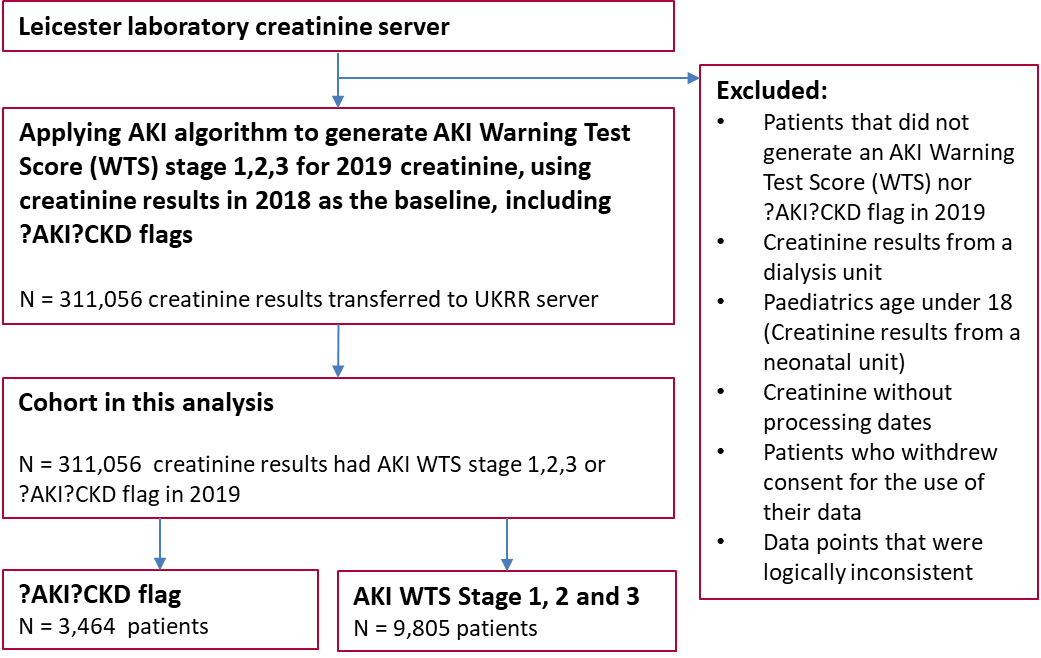


# Supplementary 2 Patient classification following ?AKI?CKD warning flags

We developed a sequential serum creatinine-based algorithm to classify patients with concurrent ?AKI?CKD warning flags.

The clinical pathway for tracking the outcome through serum creatinine blood test results over three time periods: 0-14 days, 15-90 days, and 91-365 days was illustrated in **Figure 1a and 1b**. There was a total of 3,464 patients with ?AKI?CKD warning flags. The pathway branches based on subsequent serum creatinine results:

**At 14 days:** 27.7% of patients had a serum creatinine test, with 12.2% showing minor delay in AKI detection.

**At 90 days**: 47.9% of tested patients were evaluated for serum creatinine level changes, leading to two main outcomes:

- Probable CKD (41.2% of cases)
- Delayed AKI detection/Recovered AKI (5.1% of cases)

**At 365 days:** The remaining patients were tracked for further serum creatinine results, with some showing stable results (18.2%) and others having no further testing (32%).

Patients were classified into groups according to subsequent serum creatinine results, using a consistent biochemical approach to distinguish between acute kidney injury (AKI) and chronic kidney disease (CKD). This method ensures objectivity and reproducibility in patient classification, as both AKI and CKD require laboratory confirmation according to established diagnostic criteria. Survival data were incorporated into the flow chart as supporting evidence to help validate the sequential serum creatinine-based classification algorithm, distinguishing probable AKI from probable CKD.

The flow diagram (Figure 1a 1b) uses colour coding (pink for ?AKI, yellow for ?CKD, and grey for no further results) to distinguish between different patient pathways and outcomes, which is consistent with the pie chart. The pie chart summarises the categorisation of patients following the index event based on available follow-up data.

A distinct segment of the chart represents the “Early mortality—died before follow-up” group. These patients died before repeat serum creatinine testing could be performed. While early mortality in this context may suggest the possibility of underlying AKI, we are unable to confirm this diagnosis due to the absence of follow-up biochemical data. To maintain consistency and scientific rigor, we did not classify these patients as AKI, as doing so would be speculative without laboratory evidence. Instead, they are presented as a separate subgroup to transparently acknowledge this limitation and highlight the potential clinical significance of early mortality in this setting.

# Supplementary 3 Patients demographics by ?AKI?CKD warning flags and AKI stage

The demographic characteristics of patients with ?AKI?CKD warnings (n=3,464) and AKI warning score alerts (n=9,805) are summarised in Table 1. A male predominance was observed in the suspected ?AKI?CKD group, particularly among suspected CKD cases (77%). In contrast, the AKI group showed more variation by stage, with females predominating in Stage 1 and males in Stage 3. Both groups showed a concentration of older adults, particularly those aged 75–84. A socioeconomic gradient was also noted in the suspected ?AKI?CKD group, with higher representation in less deprived areas. Seasonal variation was modest, though Stage 3 AKI peaked in winter and suspected CKD in summer.

Area-level deprivation was measured using the English Index of Multiple Deprivation (IMD) 2015, a composite small-area measure based on income, employment, education, health, crime, housing and living environment. IMD ranks for lower-layer super output areas were grouped into national quintiles, from quintile 1 (most deprived) to quintile 5 (least deprived). Deprivation quintile and calendar quarter of index date were included as categorical covariates in all models.(GOV.UK 2015)

# Supplementary 4 Survival

The following Table presents 90-day and 1-year survival outcomes for patients with AKI warning test score (WTS) alerts and those flagged as ?AKI?CKD, stratified by AKI severity (Stages 1–3) and clinical classification (probable AKI, probable CKD, or no follow-up serum creatinine). This table aims to validate the clinical relevance of our classification by comparing survival patterns: notably, the ?AKI group shows similar survival to Stage 1 AKI patients at 90 days and identical survival at 1 year, supporting the plausibility of this grouping. To minimise the influence of regional variation in outcomes, only patients from Leicester AKI WTS in 2019 were included in this comparison.

### Table S4. Survival at 90 days and 1 year by AKI Warning Test Score (WTS) stage and ?AKI?CKD warnings classification.

|  | **At 90 days** | | | | **At 1 year** | | | |
| --- | --- | --- | --- | --- | --- | --- | --- | --- |
| **Patients received AKI WTS** | **N Alive** | **Alive %** | **N Died** | **Died %** | **N Alive** | **Alive %** | **N Died** | **Died %** |
| AKI WTS stage 1 | 5884 | 84 | 1140 | 16 | 5084 | 72 | 1940 | 28 |
| AKI WTS stage 2 | 1103 | 66 | 579 | 34 | 926 | 55 | 756 | 45 |
| AKI WTS stage 3 | 730 | 66 | 369 | 34 | 584 | 53 | 515 | 47 |
| **Total AKI WTS stage 1,2,3** | 7717 | 79 | 2088 | 21 | 6594 | 67 | 3211 | 33 |
| **Patients had ?AKI?CKD warnings** | |  |  |  |  |  |  |  |
| ?AKI | 240 | 81 | 55 | 19 | 213 | 72 | 82 | 28 |
| ?CKD | 1965 | 95 | 94 | 5 | 1807 | 88 | 252 | 12 |
| No further creatinine | 1039 | 94 | 71 | 6 | 987 | 89 | 123 | 11 |
| **Total ?AKI?CKD** | 3244 | 94 | 220 | 6 | 3007 | 87 | 457 | 13 |

# Supplementary 5 Hospitalisation

### Table S5a Hospitalisation rates by demographic and clinical characteristics at 3 time points (at flag, within 14 days, and within 90 days) among patients flagged as ?AKI/?CKD, categorised into ‘probable AKI’, ‘probable CKD’, or ‘no further creatinine result’ using the algorithm described in Supplementary Figure S2

|  | **?AKI – probable AKI** | | | | **?CKD – probable CKD** | | | | **No further creatinine results** | | | | |
| --- | --- | --- | --- | --- | --- | --- | --- | --- | --- | --- | --- | --- | --- |
|  |  | **Hospitalised at time of ?AKI?CKD warning** | **Hospitalised within 14 days** | **Hospitalised within 90 days** |  | **Hospitalised at time of ?AKI?CKD warning** | **Hospitalised within 14 days** | **Hospitalised within 90 days** |  | **Hospitalised at time of ?AKI?CKD flag** | **Hospitalised within 14 days** | **Hospitalised within 90 days** |  |
|  | **N** | **%** | **%** | **%** | **N** | **%** | **%** | **%** | **N** | **%** | **%** | **%** |  |
| **Sex** |  |  |  |  |  |  |  |  |  |  |  |  |  |
| F | 92 | 64 | 86 | 88 | 527 | 13 | 26 | 36 | 228 | 12 | 17 | 20 |  |
| M | 203 | 52 | 77 | 79 | 1,531 | 16 | 26 | 35 | 881 | 8 | 11 | 14 |  |
| Missing | 3 | 67 | 67 | 67 | 6 | 17 | 17 | 17 | 6 | 0 | 0 | 0 |  |
| **Age** |  |  |  |  |  |  |  |  |  |  |  |  |  |
| 18 to 39 | 29 | 62 | 79 | 79 | 89 | 25 | 43 | 49 | 85 | 27 | 32 | 33 |  |
| 40 to 64 | 88 | 48 | 72 | 73 | 401 | 17 | 27 | 36 | 219 | 8 | 11 | 16 |  |
| 65 to 74 | 59 | 51 | 76 | 78 | 465 | 14 | 24 | 33 | 225 | 8 | 12 | 15 |  |
| 75 to 84 | 63 | 65 | 87 | 94 | 586 | 12 | 23 | 33 | 310 | 5 | 9 | 11 |  |
| 85 or over | 56 | 61 | 89 | 89 | 518 | 16 | 27 | 38 | 271 | 9 | 10 | 13 |  |
| **Season** |  |  |  |  |  |  |  |  |  |  |  |  |  |
| 1. Spring | 72 | 64 | 88 | 89 | 467 | 14 | 25 | 34 | 207 | 15 | 18 | 20 |  |
| 2. Summer | 81 | 59 | 77 | 78 | 568 | 15 | 24 | 34 | 333 | 9 | 13 | 14 |  |
| 3. Autumn | 79 | 47 | 76 | 77 | 491 | 16 | 27 | 37 | 340 | 7 | 9 | 13 |  |
| 4. Winter | 63 | 54 | 81 | 86 | 533 | 15 | 28 | 37 | 230 | 5 | 11 | 15 |  |
| **Deprivation quintile** |  |  |  |  |  |  |  |  |  |  |  |  |  |
| 1 - Most deprived | 42 | 52 | 86 | 86 | 263 | 22 | 32 | 42 | 143 | 16 | 18 | 20 |  |
| 2 | 63 | 56 | 78 | 81 | 417 | 12 | 22 | 32 | 198 | 12 | 16 | 18 |  |
| 3 | 63 | 57 | 76 | 79 | 364 | 16 | 28 | 36 | 183 | 5 | 10 | 14 |  |
| 4 | 66 | 52 | 80 | 82 | 505 | 15 | 25 | 36 | 300 | 9 | 12 | 15 |  |
| 5 - Least deprived | 58 | 62 | 83 | 84 | 504 | 15 | 26 | 35 | 280 | 5 | 8 | 11 |  |
| Missing | 3 | 67 | 67 | 67 | 6 | 17 | 17 | 17 | 6 | 0 | 0 | 0 |  |
|  |  |  |  |  |  |  |  |  |  |  |  |  |  |
| **Total** | 295 | 56 | 80 | 82 | 2,059 | 15 | 26 | 36 | 1,110 | 9 | 12 | 15 |  |

### Table S5b. Hospitalisation rates at the time, 14 days and 90 days following AKI WTS or ?AKI?CKD warning classification

|  |  | **At time of AKI WTS** | **Within 14 days** | **Within 90 days** |
| --- | --- | --- | --- | --- |
| **Patients received AKI WTS** | **N** | **%** | **%** | **%** |
| AKI WTS stage 1 | 7,024 | 60 | 72 | 78 |
| AKI WTS stage 2 | 1,682 | 69 | 89 | 92 |
| AKI WTS stage 3 | 1,099 | 62 | 87 | 92 |
| **Total AKI WTS stage 1,2,3** | 9,805 | 62 | 77 | 82 |
| **Patients had ?AKI?CKD warnings** |  |  |  |  |
| ?AKI | 295 | 56 | 80 | 82 |
| ?CKD | 2,059 | 15 | 26 | 36 |
| No further creatinine | 1,110 | 9 | 12 | 15 |
| **Total ?AKI?CKD** | 3,464 | 17 | 26 | 33 |

Hospitalisation rates varied by AKI WTS stage and by classification group from the ?AKI?CKD algorithm. Patients with AKI WTS stage 1 had a hospitalisation rate of 60% at the time of alert, rising to 78% by 90 days. Note that our data did not distinguish between index admissions, readmissions, or first-time admissions post-flag. These figures were broadly comparable to those with a ?AKI flag, who had a slightly lower rate of hospitalisation at the time of flagging (56%) but nearly identical cumulative 90-day hospitalisation (82%). This similarity supports the clinical plausibility of the ?AKI classification, though the slightly lower early hospitalisation may reflect differences in acuity, timing of presentation, or smaller sample size in the ?AKI group. In contrast, patients flagged as ?CKD had markedly lower hospitalisation rates throughout (15% at flag, 36% by 90 days), suggesting a less acute or more chronically stable profile. Hospitalisation rates in the “no further creatinine” group were lowest overall, consistent with a lower-risk or less actively managed subgroup.

### Table S5c Hospital status at ?AKI?CKD flag and within 14 days, by algorithm-based ?AKI?CKD warning/flag classification

|  |  | **In hospital at warning/flag** | **Out of hospital at warning/flag** | |
| --- | --- | --- | --- | --- |
| **Classification** | **N patients** | **N (%)** | **Admitted within 14 days N (%)** | **Not admitted within 14 days N (%)** |
| ?AKI | 295 | 165 (29%) | 71 (21%) | 59 (2%) |
| ?CKD | 2,059 | 312 (54%) | 223 (67%) | 1,524 (60%) |
| No further creatinine | 1,110 | 96 (17%) | 39 (12%) | 975 (38%) |
| Total | 3,464 | 573 (100%) | 333 (100%) | 2,558 (100%) |

# References:

GOV.UK. (2015). "English indices of deprivation 2015." from Available from: gov.uk/government/statistics/english-indices-of-deprivation-2015.

NHS Digital. (2023). "Linked HES-ONS mortality data." from <https://digital.nhs.uk/data-and-information/data-tools-and-services/data-services/linked-hes-ons-mortality-data>.

NHS England (2014) "Algorithm for detecting Acute Repeat Kidney Injury (AKI) based on serum creatinine changes with time." Members of the consensus group whose names and meeting report can be accessed on: <http://www.acb.org.uk/docs/E-Alerts_for_AKI_meeting_statement>.

NHS England (2014) "NHS England patient safety alert." DOI: <https://www.england.nhs.uk/wp-content/uploads/2014/03/psa-med-error.pdf>.
